# Effects of Early and Current Environmental Enrichment on Behavior and Growth in Pigs

**DOI:** 10.3389/fvets.2020.00268

**Published:** 2020-06-04

**Authors:** Lu Luo, Inonge Reimert, Anouschka Middelkoop, Bas Kemp, J. Elizabeth Bolhuis

**Affiliations:** Adaptation Physiology Group, Department of Animal Sciences, Wageningen University & Research, Wageningen, Netherlands

**Keywords:** behavior, early life, environmental enrichment, feed intake, growth, pigs

## Abstract

Enriched environments are known to beneficially affect the behavior of pigs, as compared with barren pens. The influence of enrichment may, however, depend on pigs' early life housing experiences. The aim of this study was to investigate the long-term effects of early and later life environmental enrichment on behavior and growth in pigs with different coping styles. Pigs were housed in either barren pens or in larger pens enriched with rooting substrates from birth, and half of them experienced a housing switch, i.e., a loss or gain of enrichment, at 7 weeks of age, creating four treatment groups. Home pen behavior and body weight were recorded until 19 weeks of age. Pigs were classified as reactive or proactive based on a backtest at 2 weeks of age. Enrichment increased time spent exploring, chewing, and play and decreased oral manipulation of penmates and pen-directed exploring and chewing. Behavior of pigs that switched from barren to enriched pens or vice versa reflected not only their actual environment, but also their early life housing. As early and later life enrichment affected most behaviors in opposite directions, effects of enrichment, or lack thereof, after the switch were more pronounced in pigs that had experienced a different early life condition. For instance, pigs experiencing an upgrade from barren to enriched pens seemed to “catch-up” by showing more exploration and play. Conversely, pigs exposed to a downgrade displayed more oral manipulation of penmates than ones kept barren throughout, which particularly held for pigs with a reactive coping style. Effects of early life and current housing on several other behaviors depended on coping style too. Pigs housed in enriched conditions appeared better able to cope with weaning than barren housed pigs, as they gained more weight and had higher feed intake post-weaning. Barren housed pigs had a lower body weight than enriched pigs just before the switch, after which growth was mainly determined by actual housing, with enriched kept pigs having a higher feed intake and body weight. Thus, not only current housing conditions, but also a (mis)match with the early life environment may affect behavior and growth of pigs.

## Introduction

Pigs in intensive farming conditions are often housed in stimulus-poor, barren environments, which offer little potential to facilitate their natural species-specific behaviors. Consequently, the limited living space, and the lack of materials for foraging and exploration in barren housing conditions, are major risk factors for the development of damaging oral behaviors, such as tail biting and ear biting (1–3). Moreover, these barren conditions can also cause chronic stress in pigs, as reflected in physiological changes (4–6). In addition, barren housing conditions were found to alter immunity (7–9) and even to increase the susceptibility to lung infections (10).

According to a European Commission Directive (2001/93/EC), pigs “must have permanent access to a sufficient quantity of materials to enable proper investigation and manipulation activities,” with the intention to improve the welfare of pigs. Numerous studies have proven that, as opposed to barren housing, enrichment of the environment with such materials, such as straw bedding or peat, can reduce damaging behaviors [e.g., (11–15)], and increase play behavior in pigs [e.g., (11, 12, 16)]. Enriched housed pigs were also more active (11, 17, 18) and showed more explorative behavior (11, 19). Some studies report enhanced growth rates in pigs kept in enriched environments [e.g., (12, 20, 21)], but see Camerlink et al. (22) and Morrison et al. (23), who did not find such an effect.

Apart from the current conditions in which pigs are housed, the environment in earlier life stages could have an effect on their later life behavior and welfare. It has been shown that adverse early life experiences can have negative long-term effects on behavior, physiology, and cognition. For instance, limited space before weaning negatively influenced the social skills needed in later life in pigs (24). Furthermore, lack of enrichment in the farrowing phase can increase the risk of tail biting in later life [e.g., (25, 26)]. Piglets may direct their exploratory behavior toward their penmates (21, 27), thus posing a risk for the development of adverse behaviors that may persist into later life. Conversely, environmental enrichment may have beneficial effects on later life functioning, putatively via its effect on brain development and functioning (28–30). For instance, rearing conditions consisting of an outdoor pasture with loose housed sows in the first 6 weeks of life suppressed the development of social stress in adult life (31), and preweaning substrate enrichment, in the form of wood shavings and chopped straw, decreased the number of agonistic encounters at a later age (21). Moreover, pigs reared in an enriched farrowing pen differed from conventionally reared pigs in behavior in a spontaneous object recognition test in later life, which could be interpreted as either reduced neophobia or improved cognitive skills in the former (32).

Hence, the behavior of pigs may, apart from being affected by their current environment, also be shaped by early life experiences. On the one hand, as outlined in the foregoing, an enriched environment in early life, as opposed to barren rearing conditions, may have long-term beneficial effects, protecting animals against developing aberrant behaviors. For example, mice reared in large, enriched cages showed less stereotypic behaviors when switched to standard cages than those in standard conditions all their lives (33, 34). On the other hand, removal of enrichment in later life might also lead to behavioral changes indicative of frustration, as animals originating from an enriched environment could be less satisfied by the poor resources in barren housing conditions. In pigs, results of some studies suggest that experience of loss of enrichment could be more detrimental than housing in barren conditions throughout life. For example, Day et al. (35) found that moving pigs from straw-bedded to barren pens increased the occurrence of damaging pen-mate-directed behavior compared to pigs without experience with straw. Less is known about the influence of switching from relatively barren to enriched housing, although Bøe (36) found that pigs that were transferred from flat deck weaner pens with slatted floors to grower pens bedded with sawdust showed more rooting and chewing of the bedding than pigs originating from bedded pens. This could be interpreted as a short-term “catching up” effect indicating an increased motivation for exploration in pigs that had been thwarted in the expression of this behavior before (12). In a previous study in which pigs were exposed to a switch from barren to straw-bedded pens, or vice versa, it was concluded that in the longer term, the behavior of pigs merely reflected their actual environment, with only subtle influences of their rearing history. The dynamics in behavioral changes were not investigated in this study, however, as the behavior was scored at two time points after the switch only and summed for analysis. It is thus still largely unknown whether potential “frustration” or “catching up” effects, resulting from a change from enriched to barren housing or vice versa, are transient or may sustain, and hence affect welfare for a longer time.

The impact of the environment on behavior may differ for pigs varying in coping style, a personality trait [e.g., (11)]. Individuals with a “proactive” coping style show a more active behavioral stress response and are prone to develop routines, whereas individuals with a “reactive” coping style tend to explore novel environments for longer and are more flexible and more attentive to subtle environmental changes. Pigs with a proactive personality seem to have more trouble in adapting to an environmental change (37, 38) that could be related to their lower flexibility in behavior as compared with reactive pigs (37, 39). On the other hand, reactive pigs may be more influenced by their long-term housing environment. Several studies reported that reactive pigs were more affected by the absence or presence of enrichment than proactive pigs (37, 40, 41), although we recently found that the behavior of proactive pigs in an attention bias test was more influenced by enrichment (42). Thus, the impact of enrichment and a loss or gain of enrichment may depend on the personality characteristics of the pig under study, which may therefore be relevant to take into account.

The aim of this study was to investigate the long-term effects of early and later life environmental enrichment on behavior and growth in pigs with different coping styles. To that aim, pigs were housed in either barren or enriched housing conditions from birth, and half of them experienced a housing switch, i.e., a loss or gain of enrichment at 7 weeks. We hypothesized that the behavior of the pigs would not only be affected by their actual housing environment, but also reflect their early life environment, particularly shortly after a change in housing. We expected the negative effects of barren housing to be more pronounced in pigs reared in an enriched environment in early life, and in reactive pigs.

## Materials and Methods

The established principles of laboratory animal use and care were followed, as well as the Dutch law on animal experiments. The Animal Care and Use Committee of Wageningen University & Research approved the experiment (DEC code: 2017.W-0001.001.IvD.3).

### Animals and Housing Before the Housing Switch

Pigs (*Tempo* × *Topigs 20*) from 30 litters, equally divided over two batches, were studied in this experiment. Multiparous *Topigs 20* sows (parity mean ± SEM: 4.1 ± 0.9) were inseminated on the same day in each batch, and were housed in Carus, the animal facilities of Wageningen University & Research, Wageningen, The Netherlands, from 1 month before farrowing. One week before the expected farrowing date, they were moved to individual farrowing pens. Distribution of sows over the housing treatments for their piglets (see later) was balanced for parity and sow weight and back fat after arrival. The maximum litter size was 14, and piglets were cross fostered within treatment if litter sizes were larger than 14. Litter size at weaning (enriched: 12.4 ± 0.2, barren: 12.1 ± 0.3 piglets/litter, *n* = 368 piglets at weaning), and weaning age (enriched: 29.7 ± 0.4, barren: 29.6 ± 0.4 days) did not differ between treatments. Pigs were not tooth resected, castrated, or tail docked.

From birth till weaning, half of piglets were housed in barren (B, 8.6 m^2^) pens with a solid floor and a small slatted area for drain. The farrowing pen had a farrowing area and a free-movement area (1.85 × 1.80 m). The sow was crated from shortly before farrowing until the piglets were 4 days of age to minimize piglet crushing. After that, the sow could move from her crate (2.85 × 0.60 m) to the free movement area and back. The other half were housed in enriched (E, 17.1 m^2^) pens. These pens consisted of exactly the same barren 8.6 m^2^ area with the sow crated as described for the B pens, to which an additional 8.6 m^2^ enriched part was added that was accessible for the piglets only. This enriched part contained 1.7 kg of straw, 300 L of sawdust, and 270 L of peat as substrates on the floor. Besides, 0.8 kg of fresh straw and 40 L of fresh sawdust were added daily, and 30 L of fresh peat was added weekly in the enriched part of the pen. Additionally, two fixed objects, here referred to as toys (one chain with a ball and one chain with screws that touched floor), were placed in the B pens. In the E pens, one fixed toy (a chain with a ball) and a toy that was alternated daily and selected from four different toys were placed. B and E pens were alternated within room.

Each pen had one drinking nipple for the piglets and one for the sow. Sows were fed a standard commercial diet twice a day. From 5 days of age until day 22, some fresh creep feed (Prestarter Speen Select, AgruniekRijnvallei, Wageningen, The Netherlands) was provided for the piglets, which was mixed with the weaner feed (Speen Uniek VC, AgruniekRijnvallei) from day 23 until weaning. The room temperature was set at 25°C and was gradually decreased to 21°C over the course of 2 weeks. In the first week after birth, one heating lamp was provided in each B pen, and two lamps in each E pen. Each pen was cleaned daily and lights and a radio were on from 07:00 until 19:00 h. Even though sows were all inseminated on the same day, farrowing was spread over a number of days. Procedures and observations below were all conducted on the same day, and days of age as referred to in the paper (except for weight at the day of birth) all relate to the number of days after the expected day of farrowing.

At 13 days of age, all pigs were subjected to a backtest to assess their coping style (43, 44). Briefly, in this test, piglets are restrained in supine position for 1 min and the number and latency of escape attempts and vocalizations are recorded [see (45) for details]. Pigs were classified as relatively “high resisters” (HRs) if they struggled 2 times and vocalized at least 25 times, or struggled at least 3 times, and as “low resisters” (LRs) if they struggled 0 or 1 time, or struggled 2 times and vocalized < 25 times (9). Thus, it was not the extremes that were selected, but the population was split into two classifications. There was no effect of housing on the proportion of HR and LR pigs (data not shown).

At 28 days of age, pigs were weaned, and in total 192 pigs (96 per batch) were selected and regrouped in 32 new pens containing 6 non-littermate pigs each. All pigs were equally regrouped by taking sex (3 males and 3 females), coping style (3 HRs and 3 LRs), and body weight into account. Housing treatment (B vs. E) for each pig was kept the same as before weaning. After weaning, therefore, the pigs from B farrowing pens were moved to barren pens (5.6 m^2^), with partly solid floor and partly slatted floor. The pigs from E farrowing pens were moved to enriched pens (11.2 m^2^) with 2.5 kg of straw, 400 L of sawdust, and 360 L of peat on the floor. Additionally, 1.25 kg of fresh straw and 60 L of fresh sawdust were added daily, and 45 L of fresh peat was added weekly in the enriched pens. The toys in the barren and enriched pens were kept the same as before weaning, and from 39 days of age, enriched housed pigs received extra enrichment such as for instance, a jute sack, a rope, branches, a log of wood or an egg tray on each Monday until the end of the experiment (day 133). B and E pens were alternated within room. The housing conditions before the switch (see later) are labeled with a “1” (i.e., B1 or E1).

Each pen had one drinking nipple and pigs received standard commercial solid feed (0–10 days after weaning: Speen Uniek VC; 11–34 days after weaning: Babybiggen Uniek VC, AgruniekRijnvallei; from 35 days after weaning onwards: Start Uniek, AgruniekRijnvallei) *ad libitum*. On the weaning day, the room temperature was set at 25°C, and it was gradually decreased to 21°C over the course of 2 weeks and kept at that temperature until the end of the experiment. After weaning, one heating lamp was provided for the first 2 weeks. Lights and a radio were on from 07:00 until 19:00 h.

### Housing After the Switch

At 47 days of age, half of the groups of pigs experienced a switch in housing type, and the other half did not. All groups of pigs, including the ones that did not change housing type, were moved to new pens, and group composition remained the same. Thus, after the switch, there were four housing treatment groups: E1E2, E1B2, B1E2, B1B2, *n* = 8 pens per group (192 pigs in total), with 1 and 2 reflecting the housing conditions before and after the switch, respectively. Straw, peat, and toys were used and added as described in the foregoing, but only 30 L of sawdust was added daily in the E2 pens.

Pigs were, both before and after weaning, housed in two rooms per batch. Distribution of (early and later life) barren and enriched pens over the rooms was balanced. Part of the pigs within a pen were exposed to tests for emotional state and immunity, the results of which are published elsewhere (42, 46, 47). This was balanced for the treatments.

### Behavioral Observations

**Figure 1:**
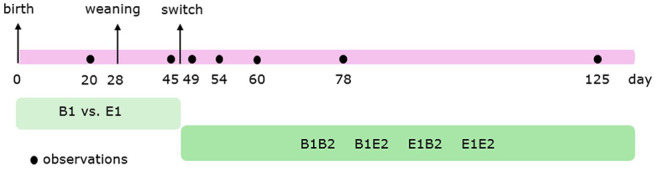
Timeline of the observations. Pigs were housed in either barren (B1) or enriched (E1) pens from birth, and half of them experienced a housing switch at 47 days of age, creating four groups: B1B2, B1E2, E1E2, and E1B2.

Figure 1 shows the timeline of the behavioural observations. Behavior (Table 1) of individual pigs before weaning, in the farrowing pens, was scored live using 4-min instantaneous scan sampling for 3 h per day at 20 and 21 days of age (in total, 6 h per pen before weaning). Behavior of individual pigs after weaning was scored live using 2.5-min instantaneous scan sampling for 6 h per day at 45 (2 days before the switch), 49 (2 days after the switch), 54 (7 days after the switch), 60 (13 days after the switch), 78 (31 days after the switch), and 125 days of age (78 days after the switch). A timeline of the observations is given in Figure 1. Observations started at 08:00 h, 09:15 h, 10:30 h, 14:00 h, 15:15 h, and 16:30 h. These procedures resulted in a total of 90 observations per pig before weaning, and 144 observations per pig per observation day after weaning. On each observation day, there was no activity in the rooms other than daily cleaning. Before weaning three, and thereafter four well-trained observers scored the behaviors from the corridor adjacent to the pens. Observers were always balanced over treatments and changed rooms every hour. Agreement between the different observers was substantial (before weaning: average Cohen's kappa (κ) = 0.75 (range: 0.71–0.79), after weaning: κ = 0.77 (0.71–0.90) (48, 49).

**Table 1:** Ethogram used for the behavioral observations.

**Behavior**	**Definition**
**Inactive behavior**	Lying on side or belly with eyes closed or open and without performing any other described behavior
**Social behavior**	Touching or sniffing any part of a penmate (= piglet or sow), including nose contact, without manipulative behaviors or massaging the udder
**Exploration**	
Substrates-directed exploring	Sniffing, nosing, licking, rooting, rubbing substrates, or scraping the substrates with one leg
Pen-directed exploring	Exploring any part of the pen (wall, floor), feeder, objects, drinking nipples and toys by sniffing, nosing, licking, rooting, or rubbing
**Chewing**	
Substrates-directed chewing	Chewing on substrates in enriched pens
Pen-directed chewing	Chewing any part of the pen (wall, mat), feeder, objects, drinking nipples, toys or ear tags, or chewing air or feces
**Manipulation**	Nibbling, sucking, chewing, or biting an ear or the tail or other part of the body of a penmate
**Play behavior**	Shaking of head while holding substrate (e.g., straw, except toy) that protrudes from mouth, or walking around the pen with substrate in mouth or lifting over substrates, shaking toy, or lifting over/pushing toys, running, jumping, rolling, turning with other pigs or individually, sometimes with gently nudging of penmates
**Aggression**	Horizontal or vertical knocking with the head or forward thrusting with the snout toward a penmate; intense mutual/individual ramming or pushing a penmate; biting a penmate, except ear or tail
**Mounting**	Standing on hind legs while having front legs on another pig's back (not the sow)
**Comfort behavior**	Rubbing body against objects or penmates, scratching body with hind leg, stretching (part of) body, or shaking body.
**Other behavior**	All other behaviors, including standing, sitting, walking without other behaviors described before, and eating, drinking, defecating and urinating

### Weight Gain and Feed Intake

Body weight of the individual pigs was measured at day 0 (within 12 h from birth), 28 (weaning), 33, and at days 46 (the day before the switch), 50 (3 days after the switch), 74 (27 days after the switch), 109 (62 days after the switch), and 130 (83 days after the switch). Feed intake was calculated per pen by registering the amount of feed given and weighing residual feed at several time points. Before weaning, residual feed was weighed at weaning on day 28. After weaning, residual feed was weighed on days 33, 47 (switch), 50 (3 days after the switch), and 133 (86 days after the switch).

### Statistical Analyses

SAS (SAS 9.4, SAS Institute Inc.) was used for all statistical analyses. Before weaning, 377 pigs were included in the home pen behavior observation analyses. From the 192 pigs selected at weaning for further study, one pig died on the day of weaning (cause unknown); therefore, there were only 5 pigs in that pen from weaning onwards. Behavioral data from 3 other pigs were missing for the last observation day at 125 days of age, as the pigs were euthanized due to health problems (lameness: n = 2 and umbilical hernia: *n* = 1). Pen-directed and substrate-directed exploring were combined as “exploring”; pen-directed chewing and substrate-directed chewing were combined as “chewing.” Pen-directed and substrate-directed exploring and chewing were also analyzed separately. Comfort behavior was rare (before weaning: 0.8%, before the switch: 0.3%, after the switch: 0.2%) and therefore not further analyzed. Defecating/urinating, eating, drinking, and walking, sitting, and standing without any other behavior were combined as “other” behavior, and were not further analyzed as well.

Assumptions of normality of error distribution and homogeneity of variance were in order, except for proportions of time spent 2 days before the switch on aggression, pen-directed chewing, social behavior, and mounting. These behaviors were therefore arcsine square root transformed to obtain normality. The same held for these behaviors and pen-directed exploration and play after the switch.

#### Behaviors

##### Before the Switch

Proportions of time spent on behaviors before weaning and before the switch were analyzed using a linear mixed model. The fixed effects of housing 1 (H1, housing before switch, B1 vs. E1), coping style (HR vs. LR), their interaction, sex and batch, and the random effect of pen nested with H1 and batch were included.

##### After the Switch

Proportions of time spent on behaviors over the 5 observation days after the switch were analyzed using a repeated linear mixed model with fixed effects of H1, housing 2 (H2, housing after switch, B2 vs. E2), coping style, observation day, their interactions, sex, the interaction of sex and observation day, and batch. Random effects of pen and animal were included to account for repeated measurements at the group (housing and batch) or individual level (sex and coping style).

#### Body Weight Gain

##### Before the Switch

Birth weight and body weight gains from birth till weaning, and during the first 5 and 18 days after weaning of the 192 selected pigs were analyzed using a linear mixed model, with H1, coping style, their interaction, sex, and batch as fixed effects, and pen nested with H1 and batch as random effect. Body weight at the beginning of each period was used as a covariate in the model, except for the analysis of body weight at birth.

##### After the Switch

Body weight gains during several periods (day 50–46, as a reflection of a potential acute effect of the switch, day 74–46, day 109–74, day 130–109, i.e., effects in different time periods post-switch, and day 130–46, the overall effect from switch to the end of the experiment) after the switch and body weight on day 130 were analyzed using a linear mixed model, with H1, H2, coping style, their interactions, sex, and batch as fixed effects, and pen nested with H1, H2, and batch as random effect. The body weight at the beginning of each period was used as a covariate in the model, except for the analysis of the body weight on day 130.

#### Feed Intake

##### Before Weaning

Feed intake (measured at pen level and expressed as kg/piglet) before weaning was analyzed using a linear model, with H1 and batch as fixed effects.

##### Before the Switch

Average daily feed intake (kg/pig/day) of different periods (day 33–28 and day 47–28, i.e., the first 5 and 19 days after weaning, respectively) before the housing switch was analyzed using a linear mixed model, with H1 and batch as fixed effects.

##### After the Switch

Average daily feed intake of different periods (day 50–47, as a reflection of the acute effect of the switch, and day 133–47, as a long-term effect) after the housing switch was analyzed using a linear mixed model, with H1, H2, their interaction, and batch as fixed effects.

Significant interactions were further investigated with *post hoc* pairwise comparisons using the difference of the least square means. Pairwise comparisons (>4 groups of means) were adjusted by Tukey corrections. Results are presented as means ± SEM.

## Results

### Behavioral Observations

#### Before Weaning

Table 2 shows the percentage of time spent on different behaviors for barren (B1) vs. enriched (E1) housed pigs at 3 weeks of age, before weaning. E1 pigs showed more exploring [*F*_(1, 28)_ = 8.96, *p* = 0.006] and chewing than B1 pigs [*F*_(1, 28)_ = 47.15, *p* < 0.001]. Exploring and chewing of B1 pigs was pen-directed only, whereas E1 pigs could also use the substrates. The pen-directed exploring [*F*_(1, 28)_ = 27.75, *p* < 0.001] and chewing [*F*_(1, 28)_ = 17.18, *p* < 0.001] of E1 pigs was lower than that of B1 pigs. Moreover, E1 pigs showed less manipulative behaviors directed at penmates [*F*_(1, 28)_ = 49.90, *p* < 0.001] and less aggression [*F*_(1, 28)_ = 5.78, *p* = 0.023]. Housing did not affect social behavior or mounting.

**Table 2:** Means ± SEM of the percentages of time spent on behaviors in pigs housed in barren and enriched housing conditions at 3 weeks of age.

**Behavior (% of time)**	**Barren**	**Enriched**	***p*-value**
Inactive	57.5 ± 2.4	51.9 ± 2.0	+
Social behavior	1.8 ± 0.2	1.4 ± 0.1	ns
Exploring^a^	7.2 ± 0.6	11.1 ± 1.1	**
Pen-directed exploring	7.2 ± 0.6	3.4 ± 0.3	***
Chewing^b^	2.8 ± 0.4	10.0 ± 1.1	***
Pen-directed chewing	2.8 ± 0.4	1.3 ± 0.1	***
Manipulation	2.4 ± 0.2	0.7 ± 0.1	***
Play	1.5 ± 0.3	2.4 ± 0.4	+
Aggression	1.2 ± 0.2	0.7 ± 0.1	*
Mounting	0.4 ± 0.1	0.4 ± 0.1	ns

a *Exploration includes pen-directed exploring and exploring the substrates*.

b *Chewing includes pen- and substrates-directed chewing*.

Significances of differences are indicated: ***p < 0.001, **p < 0.01, *p < 0.05, and ^+^p < 0.10; ns indicates non-significance.

Coping style did not affect behavior before weaning.

Females showed less aggression [F: 0.6 ± 0.1, M: 1.3 ± 0.1% of observations, *F*_(1, 343)_ = 37.24, *p* < 0.001], and mounting [F: 0.2 ± 0.04, M: 0.6 ± 0.07%, *F*_(1, 343)_ = 34.19, *p* < 0.001] before weaning than males. No other sex effects were found.

#### Before the Switch

Table 3 shows the percentage of time spent on behaviors for enriched (E1) vs. barren (B1) housed pigs at 6 weeks of age, 2 days before the switch. E1 pigs displayed less inactive behavior [*F*_(1, 29)_ = 21.89, *p* < 0.001] and more exploration [*F*_(1, 29)_ = 9.54, *p* = 0.004] and chewing [*F*_(1, 29)_ = 120.71, *p* < 0.001] than B1 pigs. Pen-directed exploration [*F*_(1, 29)_ = 59.89, *p* < 0.001] and pen-directed chewing [*F*_(1, 29)_ = 193.60, *p* < 0.001] were lower in E1 pigs than in B1 pigs. E1 pigs showed less manipulative behaviors directed at penmates [*F*_(1, 29)_ = 80.72, *p* < 0.001] and mounting [*F*_(1, 29)_ = 4.93, *p* = 0.034] than B1 pigs. Housing did not affect social behavior, play behavior, or aggression (see Table 3). Coping style only affected pen-directed exploration [HR: 4.0 ± 0.4, LR: 3.4 ± 0.3%, *F*_(1, 156)_ = 4.81, *p* = 0.030] and chewing [HR: 16.5 ± 1.2; LR: 14.9 ± 1.0%, *F*_(1, 156)_ = 4.09, *p* = 0.045].

**Table 3:** Means ± SEM of the percentages of time spent on behaviors in pigs housed in barren and enriched housing conditions 2 days before the switch (at 47 days of age).

**Behavior (% of time)**	**Barren**	**Enriched**	***p*-value**
Inactive	58.2 ± 2.3	44.7 ± 2.0	***
Social behavior	0.8 ± 0.1	0.7 ± 0.1	ns
Exploring^a^	6.1 ± 0.6	8.8 ± 0.6	**
Pen-directed exploring	6.1 ± 0.6	1.4 ± 0.1	***
Chewing^b^	7.6 ± 0.7	23.8 ± 1.3	***
Pen-directed chewing	7.6 ± 0.7	0.8 ± 0.1	***
Manipulation	2.6 ± 0.3	0.6 ± 0.2	***
Play	0.9 ± 0.2	1.4 ± 0.2	ns
Aggression	0.7 ± 0.1	0.5 ± 0.1	ns
Mounting	1.0 ± 0.1	0.5 ± 0.1	*

a *Exploration includes exploring the pen and exploring the substrates*.

b *Chewing includes pen- and substrates-directed chewing*.

Significances of differences are indicated: ***p < 0.001, **p < 0.01, *p < 0.05; ns indicates non-significance.

Females showed less aggression [F: 0.3 ± 0.1, M: 1.0 ± 0.1%, *F*_(1, 156)_ = 27.86, *p* < 0.001] and mounting [F: 0.4 ± 0.1, M: 1.0 ± 0.1%, *F*_(1, 156)_ = 18.40, *p* < 0.001] than males, but chewed more [F: 16.8 ± 1.2, M: 14.7 ± 1.0%, *F*_(1, 156)_ = 8.45, *p* = 0.004]. No other sex effects were found.

#### After the Switch

**Figure 2:**
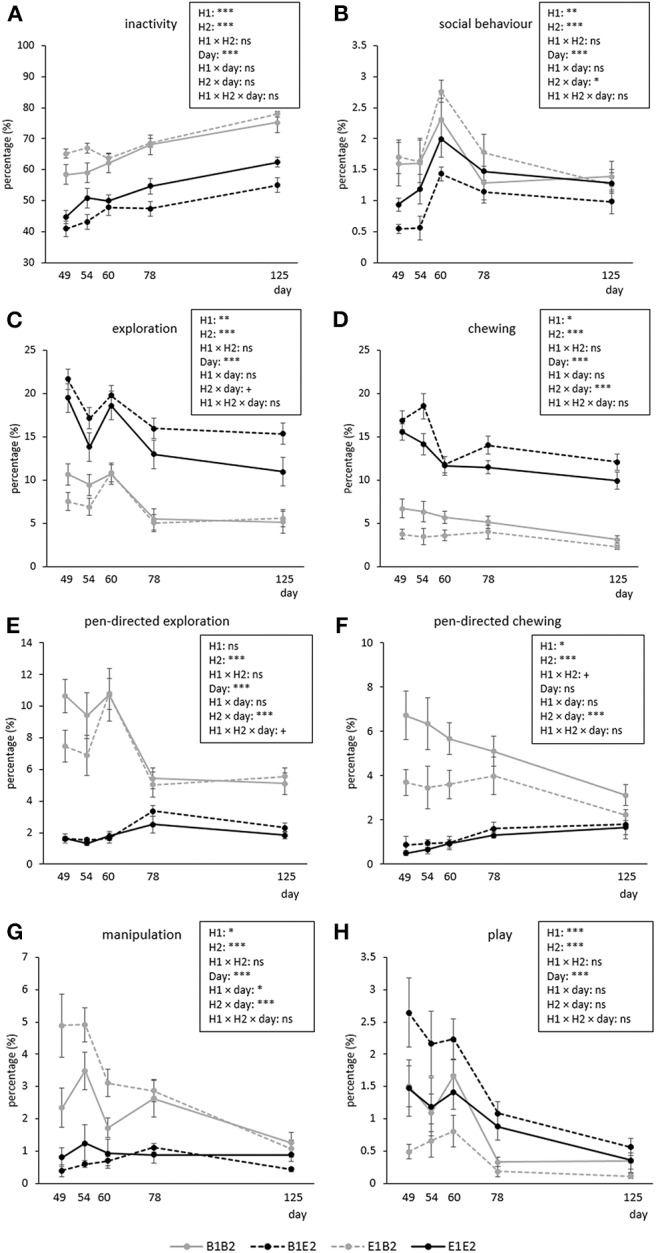
Means ± SEM of percentages of time spent on behaviors in pigs exposed to four different housing situations (*n* = 192 pigs, 32 pens, 8 pens/housing treatment). Pigs were housed in either barren (B1) or enriched (E1) pens from birth, and half of them experienced a housing switch at 47 days of age, creating four groups: B1B2, B1E2, E1E2, and E1B2. Day refers to piglet age. Significances of treatments are indicated: ***p < 0.001, **p < 0.01, *p < 0.05, and ^+^*p* < 0.10; ns indicates non-significance. H1 indicates the effect of housing before the switch (B1 vs. E1), and H2 indicates the effect of housing after the switch (B2 vs. E2). Interactions with coping style are given in Figure 3.

**Figure 3:**
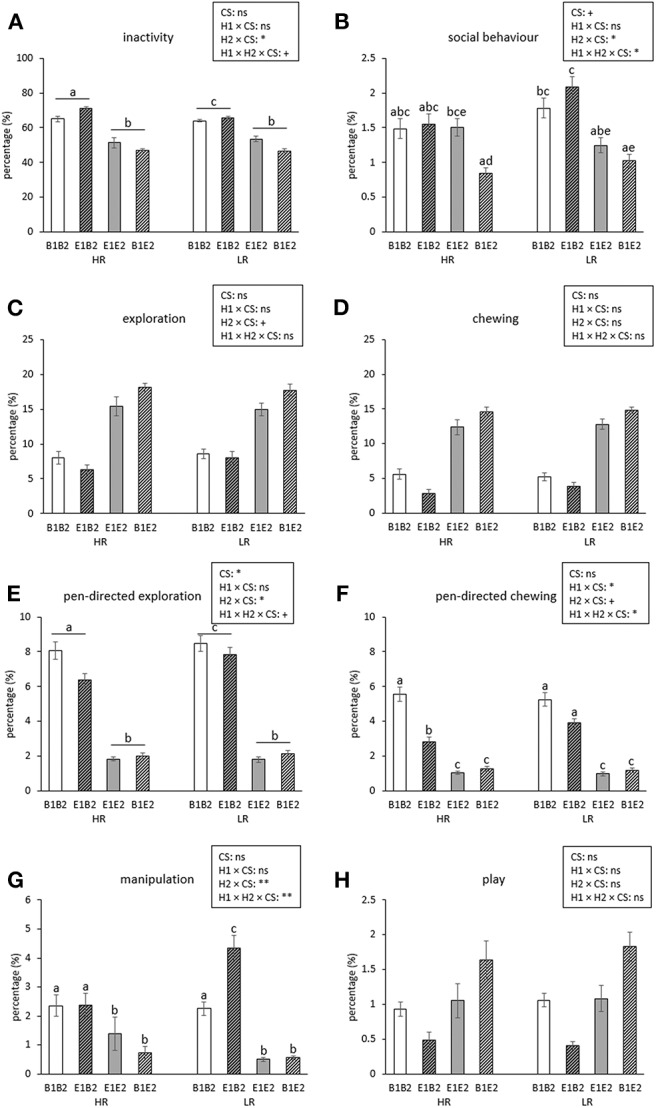
Means ± SEM of percentages of time spent on behaviors for high-resisters (HRs) vs. low-resisters (LRs) housed in four housing groups (*n* = 192 pigs, 32 pens, 8 pens/housing treatment) over 5 observation days after the switch. Means lacking a common letter differ significantly (*p* < 0.05 or less). Effects of coping style (CS) and its interactions with housing (housing before switch: H1, housing after switch: H2) are indicated: **p < 0.01, *p < 0.05, and ^+^*p* < 0.1; ns indicates non-significance. Please note: The main effects of H1, H2, their interaction, and their interactions with day can be found in Figure 2.

Figure 2 shows the time course of inactive, social, explorative, chewing, manipulative, and play behavior for the four combinations (B1B2, B1E2, E1B2, E1E2) of early life (H1, housing before the switch) and later life (H2, housing after the switch) housing. As several interactions between housing and coping style were found, which were not dependent on time (except for manipulation, see text later), these are illustrated separately in Figure 3. Aggression and mounting were mainly affected by sex and are therefore only indicated in the text.

**Inactive behavior** increased over time [day effect, *F*_(4, 112)_ = 44.69, *p* < 0.001, Figure 2A]. Pigs originating from enriched conditions (E1) were more inactive than those from early life barren housing [B1, H1 effect *F*_(1, 27)_ = 18.60, *p* < 0.001]. An opposite effect was found for H2, but the H2 effect depended on coping style [H2 × coping style interaction, *F*_(1, 154)_ = 5.02, *p* = 0.027]. B2-HR pigs were the most inactive, followed by B2-LR pigs and thereafter all E2 pigs (B2-HR: 68.1 ± 1.2, B2-LR: 64.9 ± 0.6, E2-HR: 49.2 ± 1.5, E2-LR: 50.2 ± 1.3%, all pairwise differences mentioned *p* < 0.05, Figure 3A).

**Social behavior** was affected by the H2 × day interaction [*F*_(4, 112)_ = 2.48, *p* = 0.048], with higher levels for B2 pigs than for E2 pigs on the first observation day only (Figure 2B). Social behavior was also affected by the H1 × H2 × coping style [*F*_(1, 154)_ = 4.09, *p* = 0.045] interaction (Figure 3B). Levels of social behavior were generally lowest in the pigs that switched from barren to enriched housing (B1E2) and highest in the E1B2 pigs, but the contrast with the other housing conditions depended on coping style. B1E2-HR pigs showed less social behavior than E1E2-HR pigs (*p* < 0.05), with no other pairwise differences between housing conditions for the HR pigs. More contrasts between the H1 and H2 combinations were seen in LR animals, as B1E2-LR showed lower levels of social behavior than E1B2-LR (*p* < 0.001) and B1B1-LR pigs (*p* < 0.05). Additionally, E1B2-LR pigs, apart from showing more social behavior than B1E2-LR pigs, also spent more time on this behavior than E1E2-LR pigs, *p* < 0.05, Figure 3B).

**Exploration** was affected by day [*F*_(4, 112)_ = 22.89, *p* < 0.001], with a decrease in time, except for the third observation day (Figure 2C). H1 [B1: 13.1 ± 0.4, E1: 11.2 ± 0.3%, *F*_(1, 27)_ = 10.94, *p* = 0.003] and H2 [B2: 7.7 ± 0.2, E2: 16.6 ± 0.3%, *F*_(1, 27)_ = 218.73, *p* < 0.001] affected exploration in opposite directions (Figure 3C).

Pen-directed exploration was affected by H2 × day [*F*_(4, 112)_ = 19.33, *p* < 0.001]. For E2 pigs, time spent on pen-directed exploration did not differ between days. In B2 pigs, pen-directed exploration was higher on day 60 than on day 49 and day 54, and later in life, at 78 and 125 days of age, lower than the earlier 3 days (Figure 2E). In addition, the H2 × coping style interaction [*F*_(1, 154)_ = 4.34, *p* = 0.039] affected pen-directed exploration (Figure 3E). B2-LR pigs spent more time exploring pen fixtures than B2-HR pigs, which were, in turn, followed by E2 pigs (B2-HR: 7.2 ± 0.3, B2-LR: 8.2 ± 0.3, E2-HR: 1.9 ± 0.1, E2-LR: 2.0 ± 0.1%, *p* < 0.05).

Substrate exploration in E2 pigs was affected by day [*F*_(4, 56)_ = 14.67, *p* < 0.001], with a decrease over time, except for day 60. Pigs originating from barren housing (B1E2: 15.8 ± 0.4%) spent more time on this behavior than pigs that were housed in enriched conditions throughout [E1E2: 13.4 ± 0.4%, H1-effect, *F*_(1, 13)_ = 7.13, *p* = 0.019].

**Chewing** was affected by the H2 × day interaction [*F*_(4, 112)_ = 5.30, *p* < 0.001, Figure 2D]. Time spent chewing did not change over time in B2 pigs, but in E2 pigs time spent chewing was higher on day 49 and 54 than on the days thereafter. B1 pigs showed more chewing (10.0 ± 0.3%) than E1 pigs [8.0 ± 0.3%, H1 effect, *F*_(1, 27)_ = 13.75, *p* = 0.001].

Pen-directed chewing was affected by H2 × day [*F*_(4, 112)_ = 11.05, *p* < 0.001, Figure 2F]. It increased in E2 pigs (with higher levels on day 78 and 125 than on day 49) but decreased in B2 pigs (with lower levels on day 125 than on other days). Pen-directed chewing was also affected by the H1 × H2 × coping style interaction [*F*_(1, 154)_ = 4.05, *p* = 0.046]. Pigs from E2 housing chewed less on pen-fixtures than those from B2 housing, but in HR pigs, enriched housing in early life seemed to reduce this effect, as E1B2-HR pigs chewed < E1B2-LR pigs and B1B2-HR pigs (*p* < 0.05, Figure 3F).

Substrate chewing in E2 pigs decreased from day 60 onwards [day effect, *F*_(4, 56)_ = 17.99, *p* < 0.001]. Pigs originating from barren housing (B1E2: 13.5 ± 0.4%) spent more time on chewing substrates than E1E2 pigs [11.6 ± 0.3%, H1 effect, *F*_(1, 13)_ = 6.40, *p* = 0.025].

**Manipulation** was affected by H1 × H2 × coping style × day interaction [*F*_(4, 613)_ = 3.34, *p* = 0.01]. *Post hoc* analyses showed that within HR pigs, B2-HR pigs (both B1B2 and E1B2) manipulated more than E2-HR (B1E2 and E1E2) pigs (*p* < 0.05), whereas LR pigs that switched from enriched to barren housing (E1B2-LR) showed more manipulation than all others, including HR pigs under the same circumstances (E1B2-HR) and LR pigs that were housed in barren pens throughout (B1B2-LR, *p* < 0.05, Figure 3G). On the last observation day, however, the H1 × H2 × coping style interaction [over all days: *F*_(1, 154)_ = 7.97, *p* = 0.005] had disappeared as H1 and the interaction between housing and coping style did not influence time spent on oral manipulation on that day, whereas H2 and coping style effects remained.

**Play** decreased over time [*F*_(4, 112)_ = 23.90, *p* < 0.001], and was affected by H1 and H2 in opposite directions, as B1 pigs (1.4 ± 0.1%) played more than E1 pigs [0.8 ± 0.05%, *F*_(1, 27)_ = 22.16, *p* < 0.001], whereas enrichment in later life increased play behavior [B2: 0.7 ± 0.05, E2: 1.4 ± 0.1%, *F*_(1, 27)_ = 27.38, *p* < 0.001].

**Aggression** decreased over time [day effect, *F*_(4, 112) =_ 7.23, *p* < 0.001]. In B2 pigs, aggressive behavior was higher on day 54 (1.0 ± 0.1%) than on day 78 (0.5 ± 0.1%) and 125 (0.4 ± 0.1%), and in E2 pigs aggression was higher on day 49 (1.1 ± 0.1%) than on day 125 [0.3 ± 0.1%, H2 × day effect, *F*_(4, 112)_ = 3.36, *p* = 0.012], without an H2 effect within any of the days. Time spent on aggression was also affected by coping style [HR: 0.6 ± 0.04, LR: 0.8 ± 0.04%, *F*_(1, 154) =_ 8.18, *p* = 0.005].

**Mounting** decreased over time [day effect *F*_(4, 112)_ = 6.53, *p* < 0.001], being lower on the last two observation days than on the first observation day after the switch, but this held only for B2 pigs (day 49: 0.6 ± 0.08, day 78: 0.2 ± 0.06, day 125: 0.3 ± 0.06%), whereas time spent mounting was rather constant in E2 pigs [H2 × day interaction, *F*_(4, 112)_ = 3.01, *p* = 0.021, average E2: 0.3 ± 0.03 %].

#### Sex Effects

After the switch, inactive behavior was affected by the sex × day interaction [*F*_(4, 613)_ = 6.49, *p* < 0.001], with females being more inactive at 60 days of age than males (*p* < 0.05, Supplementary Table 1). Females showed less social behavior [F: 1.3 ± 0.1, M: 1.6 ± 0.1%, *F*_(1, 154)_ = 15.28, *p* = 0.001], explored less [F: 11.8 ± 0.3, M: 12.4 ± 0.3%, *F*_(1, 154)_ = 5.09, *p* = 0.026], and showed less substrate exploration [F: 14.0 ± 0.4, M: 15.2 ± 0.4%, *F*_(1, 76)_ = 4.99, *p* = 0.028] than males. Time spent chewing [*F*_(4, 613)_ = 2.73, *p* = 0.028], chewing substrates [*F*_(4, 403)_ = 2.93, *p* = 0.021], and oral manipulation [*F*_(4, 613)_ = 3.75, *p* = 0.005] were affected by the sex × day interaction, without significant pairwise differences (Supplementary Table 1). Females played less than males on day 60 [sex × day effect, *F*_(4, 613)_ = 7.13, *p* < 0.001, Supplementary Table 1]. The sex × day effect [*F*_(4, 613)_ = 9.69, *p* < 0.001] found for aggression indicated that females spent less time on aggressive behaviors than males on all observation days, except the last one (Supplementary Table 1). Males mounted more (0.5 ± 0.04%) than females [0.1 ± 0.02%, *F*_(1, 154)_ = 63.46, *p* < 0.001]. No effect of sex or its interaction with day was found on pen-directed exploration and pen-directed chewing.

### Body Weight Gain and Feed Intake

#### Before the Switch

The body weights of barren pigs and enriched pigs at birth were not different (B1: 1.5 ± 0.03, E1: 1.5 ± 0.02 kg). From birth until weaning at day 28, E1 pigs gained more weight than B1 pigs [E1: 7.5 ± 0.1, B1: 6.9 ± 0.2 kg, *F*_(1, 29)_ = 6.62, *p* = 0.015]. Besides, LR pigs tended to gain more than HR pigs from birth until weaning [LR: 7.4 ± 0.2 kg, HR: 7.0 ± 0.2, *F*_(1, 156)_ = 3.34, *p* = 0.069]. Creep feed intake before weaning (days 5–28) tended to be higher in E1 pigs (0.42 ± 0.12 kg/pig) than in B1 pigs [0.27 ± 0.09 kg/pig, *F*_(1, 27)_ = 3.41, *p* = 0.076].

Enriched pigs also gained more weight during the first five [E1: 1.2 ± 0.1, B1: 0.8 ± 0.1 kg, *F*_(1, 29)_ = 21.47, *p* < 0.001] and 18 days [E1: 9.1 ± 0.3, B1: 7.6 ± 0.2 kg, *F*_(1, 29)_ = 26.64, *p* < 0.001] after weaning. Average daily feed intake during the first five [E1: 0.34 ± 0.02, B1: 0.28 ± 0.02 kg/pig/day, *F*_(1, 29)_ = 14.47, *p* < 0.001] and 19 days [E1: 0.61 ± 0.01, B1: 0.55 ± 0.01 kg/pig/day, *F*_(1, 29)_ = 11.71, *p* = 0.002] after weaning was also higher for E1 pigs than B1 pigs. As a result, E1 pigs were heavier (18.1 ± 0.3 kg) just before the switch than B1 pigs [16.1 ± 0.3 kg, *F*_(1, 29)_ = 24.85, *p* < 0.001].

#### After the Switch

Table 4 shows the growth in the four housing treatment groups after the switch. With body weight at the start of each period as a covariate in the model, no significant housing effects were found for growth shortly after the switch (days 46–50). Growth over the first 28 days after the switch (days 46–74) tended to be higher in pigs in enriched housing after the switch [H2 effect, *F*_(1, 7)_ = 3.65, *p* = 0.067]. Growth between days 74 and 109 was not influenced by housing, but body weight gain from day 109 to day 130 was affected by H1 [E1: 22.6 ± 0.6, B1: 24.4 ± 1.0 kg, *F*_(1, 27)_ = 4.79, *p* = 0.037], and H2 [E2: 24.4 ± 1.0, B2: 22.6 ± 0.6 kg, *F*_(1, 27)_ = 6.03, *p* = 0.021] in opposite directions. Overall, the body weight gain from 1 day before the switch (day 46) until the end of the experiment (day 130) was higher for E2 (86.0 ± 1.0 kg) than for B2 pigs [81.4 ± 0.9 kg, H2 effect, *F*_(1, 27)_ = 11.86, *p* = 0.002]. As a result, on day 130, E2 pigs were heavier (103.4 ± 1.0 kg) than B2 pigs [98.2 ± 0.9 kg, *F*_(1, 27)_ = 18.66, *p* = 0.002].

**Table 4:** Means ± SEM of body weight gains (kg) in pigs housed in four housing groups after the housing switch (at 47 days of age).

**Period**	**B1B2**	**B1E2**	**E1B2**	**E1E2**	**H1**	**H2**
Day 46–50	2.5 ± 0.2	2.6 ± 0.1	2.6 ± 0.3	2.8 ± 0.2	Ns	ns
Day 46–74	20.5 ± 0.8	21.7 ± 0.6	22.1 ± 0.9	23.4 ± 0.4	Ns	+
Day 74–109	37.5 ± 1.1	38.8 ± 1.6	37.3 ± 0.9	39.4 ± 1.3	Ns	ns
Day 109–130	23.4 ± 1.0	25.4 ± 1.8	21.9 ± 0.7	23.4 ± 1.0	*	*
Day 46–130	81.5 ± 1.3	85.9 ± 1.5	81.3 ± 1.2	86.1 ± 1.3	Ns	**

H1 indicates the effect of housing before the switch, and H2 indicates the effect of housing after the switch. No H1 × H2 interactions were found. Significances of differences are indicated: **p < 0.01, *p < 0.05, and ^+^p < 0.10; ns indicates non-significance.

There were no coping style effects on body weight gain.

No sex effect was found on body weight gains before the housing switch. After the switch, females gained less weight than males in all periods, except the first days after the switch (data not shown). Their growth from the switch until day 130 therefore also diverged [F: 81.9 ± 0.9, M: 85.4 ± 0.7 kg, *F*_(1, 150)_ = 12.71, *p* < 0.001] and at the end of the experiment on day 130 females were lighter than males [F: 98.9 ± 1.0, M: 102.6 ± 0.8 kg, *F*_(1, 151)_ = 8.41, *p* = 0.004].

During the first 3 days after the switch (days 47–50), the average daily feed intake was affected by H2, with higher levels in E2 pigs (1.6 ± 0.2 kg/pig/day) than in B2 pigs [1.1 ± 0.1 kg/pig/day, *F*_(1, 27)_ = 7.07, *p* = 0.013], but no H1 effect or H1 × H2 interaction was found. In the period from the switch to the end of the experiment (days 47–133), the average daily feed intake was also affected by H2 [E2: 2.1 ± 0.04, B2: 2.0 ± 0.03, kg/pig/day, *F*_(1, 27)_ = 10.34, *p* = 0.003] only.

## Discussion

In this study, pigs were exposed to an either barren or enriched (with substrates, toys, and more space) early life environment, after which half of them experienced a switch in housing conditions from barren to enriched or vice versa at 7 weeks of age. This allowed us to study the impact of early life and current housing, as well as that of an upgrade or downgrade shift in environmental conditions on their behavior and body weight development up until 19 weeks of age. We found that the behavior of the pigs after the switch not only reflected their actual environment but was also influenced by early life housing conditions. Generally, effects of housing conditions after the switch seemed more pronounced in pigs that had experienced a different early life condition. Some of the effects of early life or current housing depended on the coping style of the pigs.

### Effect of Housing and Coping Style

#### Effects of Enrichment Before the Switch

Enrichment of the farrowing pen in the form of rooting substrates and alternating toys and extra space reduced manipulative behavior directed to penmates, aggression, inactivity, increased exploration, and chewing and tended to increase play behavior before weaning. Similar results were found on day 45 (2 days before the housing switch), except that on this day barren and enriched pigs no longer differed in time spent on play and aggression. The impact of an enriched environment reported here is largely in line with other studies using substrates and extra space (17, 20, 50) or substrates only (11, 12, 51) as enrichment. As expected, barren housed pigs spent more time on exploring their pen and on chewing on pen fittings or air and performed more oral manipulative behavior directed at their penmates than enriched pigs. This suggests that pigs without suitable rooting materials direct more explorative behavior to their pen and penmates (52). Nevertheless, total time spent on exploration and chewing were still lower for barren housed pigs, likely because the pen fittings and bare floor did not meet the criteria important to make exploration worthwhile, as pigs prefer “chewable,” “destructible,” “rootable,” and “deformable” materials (53). It has been suggested that lowered levels of activity and exploration may reflect an apathetic response to an aversive environment and thus may indicate poor welfare (54–56).

Enrichment reduced aggression before, but not after weaning. Beattie and O'connell (17) found a reduction in aggression in pigs provided with extra space and rooting substrates, whereas studies comparing similar sized pens with or without rooting substrates reported either no effect (11, 51), a decrease (57) or an increase in aggression (58, 59). Thus, effects of enrichment on aggression seem inconsistent. This might be related to the type of enrichment provided, the timespan since application of enrichment, and the social setting, e.g., whether and when new groups were formed.

Play behavior is known to peak between 2 and 6 weeks of age in pigs and decreases thereafter with age (60, 61). Enriched housed pigs tended to spend more time on play behavior before weaning and significantly played more after the switch in housing at 7 weeks. Also others reported more play behavior in pigs kept in enriched as compared with barren pens (11, 12, 16, 32, 62, 63). It has been put forward that several types of play behavior are suppressed in adverse physical and environmental conditions (64) and following severe or prolonged negative emotions (65, 66). Some studies also suggest that a reduction in play behavior in early life may negatively affect the development of behavior and brain, leading to poorer social skills and behavioral flexibility, and therefore may have a long-term effect on adaptive capacity and welfare (67–69).

In this study, we found that enriched housed pigs were better able to cope with weaning transition, as they gained more weight and had a higher feed intake during the first 5 and 18 days after weaning. This could be related to the finding that enriched pigs also tended to eat more before weaning. Preweaning consumption of creep feed is known to stimulate post-weaning feed intake and thereby reduces the weaning-related growth check and other problems [e.g., (70–72)]. The better post-weaning performance of enriched pigs may, apart from resulting from an enhanced development of feeding-related behaviors, also reflect increased adaptability of piglets reared in enriched conditions to stressful processes such as weaning (63, 73). However, it has been shown that post-weaning environmental enrichment alone, irrespective of preweaning housing, also improves performance and health of newly weaned piglets (73), which may be mediated by preserving gut functioning [e.g., (74, 75)], either through intake of substrates or through stress reduction [e.g., (51, 76)].

#### After the Housing Switch

The behavioral development of the pigs from 7–19 weeks of age was characterized by an increase in inactivity and a decrease in most of the active behaviors, in accordance with previous studies [e.g., (11, 52)]. This is likely related to the increased age and weight and reduced space available per pig over time (52). Effects of the current environment after 7 weeks were largely comparable to those before the switch. Pigs kept in enriched pens spent more time on exploration, chewing, and play behavior, whereas barren housed pigs were less active and showed more social behavior, oral manipulation of penmates and pen-directed exploration and chewing. The housing effect on social behavior, which was not seen before 7 weeks of age, might partly reflect an increased inspection of penmates' bodies in barren pens due to the absence of rooting substrates.

Animals experiencing a switch in environment thus generally adjusted their behavior to the current housing. Housing before the switch at 7 weeks of age, however, still exerted an influence on all behaviors with the exception of pen-directed exploration, demonstrating an interplay of past experiences and the current housing environment. Notably, for all behaviors except pen-directed chewing, the effect of housing before the switch was opposite to that of housing after the switch. Adding up the early life and current housing effects, therefore, meant that the switched groups of pigs (E1B2 and B1E2) differed the most in their behavior. For instance, both play and chewing were seen most often in pigs that switched from barren to enriched housing, and least in pigs experiencing a downshift from enriched to barren pens. Also, pigs that originated from barren housing spent more time on substrate-related behaviors after the switch than pigs kept in enriched housing throughout. Correspondingly, manipulative, social, and inactive behaviors were highest in E1B2 pigs and lowest in B1E2 pigs, albeit in a coping style-dependent way (see later). Thus, a switch in housing conditions seemed to enlarge the effect of current housing on behavior, implying a larger impact of presence or absence of enrichment in pigs with an opposite rearing history. It has been suggested that loss of enrichment, i.e., switching from enriched to barren housing, is more detrimental than barren housing throughout life (21, 77), which seems to be demonstrated in this study as well. Conversely, pigs that changed from barren to enriched housing seemingly tried to “catch-up,” an effect retaining for months. This may reflect an increased motivation for exploration and play as the expression of these behaviors were hampered because of lack of space and rooting materials earlier in life [(78), cited in (11)]. This “catching up” effect does not imply that lack of enrichment in early life can be made up for by later life enrichment, but rather that enrichment is important for pigs in all life stages.

The opposite effect of early life housing as compared with current housing, however, was not found for pen-directed chewing as B1B2 pigs spent more time on this behavior than E1B2 pigs, although the effect was coping style-dependent (see later). It could be that the E1B2 pigs, as they were used to more attractive substrates in early life, were not satisfied by chewing on pen fixtures, and therefore turned to more nosing and oral manipulation of their penmates, especially in the first weeks after the housing switch. It thus seems that there is no “protective” effect of early enriched housing on the development of damaging behaviors, but rather a downshift (“frustration”) effect was shown. Notably, early life housing had rather long-term effects on most of the behaviors, as there was no interaction between early life housing and day, except for manipulation, where the early life effect had disappeared by day 125. Another study, however, found that the influence of early life rearing history on behavior of pigs was smaller and merely overruled by current housing conditions (12). The different results in the current study could be related to a larger contrast in housing, as enrichment here not only encompassed straw, but also other substrates and extra space, or to more observation weeks, and/or a larger sample size.

Even though we found long-term effects of the housing switch on behavior, it is unknown whether the interplay between early life and current housing also impacted the affective states of the pigs for such a long time. Douglas et al. (79) reported more pessimistic judgement biases in pigs that had switched from enriched to barren pens as compared to pigs that initially were housed barren. However, in their study pigs were tested only shortly (up to 7 days) after the change in housing. We found that pigs experiencing an upgrade in the quality of their environment played most often, whereas pigs that were subjected to a downgrade from enriched to barren housing conditions showed the least play. Play behavior has been linked to positive emotions [review in (80)], and therefore, the findings in this study may suggest that a gain or loss of enrichment exerts a long-term impact on the affective state of pigs. However, other studies do not support this suggestion. We subjected part of the pigs to an attention bias test (42) and successive negative contrast test (46) to assess a potential long-term impact of their housing (history) on affective state, but failed to show the expected effects of the negative or positive housing switch on the responses of pigs in these tests. Rather, the successive negative contrast test suggested a “protective” effect of early life enrichment irrespective of the current environment, as pigs reared in enriched pens were less susceptible to reward loss (46). In the attention bias test, no early life effects were found, whereas the impact of current housing was opposite to expectations, which might be due to the test circumstances (42), and found only for pigs with a proactive coping style. Thus, further research is needed to evaluate whether a change from enriched to barren housing conditions and vice versa not only affects pigs' behavior, but also their affective state in the long run.

#### Coping Style Effects

Effects of enrichment on several behaviors were found to be coping style-dependent, even though we did not select extremes for our study, but labeled each pig as either LR (reactive) or HR (proactive) based on a single backtest early in life. Barren housing increased inactivity in both types of pig, but more so in the HR pigs, whereas pen-directed exploration was enhanced in the barren housed LR pigs. Also the impact of a switch in housing conditions on some behaviors varied for both types of pig, as social behavior, pen-directed chewing and manipulation were affected by the interaction between early life housing, current housing and coping style. LR pigs that experienced a downgrade in housing from enriched to barren pens showed more pen-directed chewing and oral manipulation of penmates than their HR counterparts. The strong increase in these oral damaging behaviors in LR pigs that had switched from enriched to barren housing, exceeding the levels of pigs kept in barren pens throughout, might indicate that LR pigs were most affected by the loss of exploratory stimuli and space. Also in other studies LR pigs showed more oral manipulation than HR pigs, especially when housed barren (11), and, in contrast to the current study, even more so when also reared in barren pens (12). It has been suggested that LR pigs have a higher motivation to explore their surroundings than HR pigs (81), and therefore show more manipulative behavior directed at pen and penmatess when substrates are not available (11). The effects of rooting substrates on the occurrence of gastric lesions (12), immune reactivity (41), and response to novelty (37) were also larger in LR than in HR pigs, suggesting that reactive pigs are more susceptible to (lack of) enrichment. In support of this, Asher et al. (40) found that reactive pigs in barren pens were more pessimistic and those in enriched pens were more optimistic in a cognitive bias test, whereas the response of proactive pigs was less affected by their housing environment. It should be noted, though, that the housing effect on the behavioral response in an attention bias test, to which a subset of the pigs of the current study was exposed, was stronger for proactive pigs (42), which is not in line with other findings.

Irrespective of housing treatments, LR pigs spent more time on aggressive behaviors than HR pigs after the housing switch. This is in contrast with other studies describing higher levels of aggression in HR pigs, both in stable groups (11, 12) and when mixed (82), or reporting no differences between LR and HR pigs (83). These inconsistent results could be related to the finding that, rather than differing in level of aggression *per se*, the types of pig may vary in how flexibly they can attune their aggressive behavior (39, 45).

### Body Weight Gain

In the present study, enriched housed pigs had higher body weight gains than barren housed pigs, both before and after the switch, as well as a higher feed intake. At the end of the experiment (around 19 weeks of age), pigs kept in enriched pens from the switch at 7 weeks onwards were heavier, irrespective of their early life housing conditions. This means that pigs originating from barren housing, even though they had a lower body weight before the switch than enriched pigs, caught up after being placed in enriched pens. The reverse was found in pigs from enriched housing that changed to barren pens. This is illustrated by the growth from day 109 to day 130, which was affected by both early life and current housing, but in opposite directions. As a result, B1E2 pigs gained the most weight, and E1B2 the least. Also other studies report that environmental enrichment can improve the growth of pigs [e.g., (17, 84)]. It has been shown that oral manipulation can negatively affect growth (85), which could be either due to stress in the victims, or because the wounds caused by oral manipulation may lead to inflammation and infection (86). In our study, barren pigs indeed showed more tail lesions following oral manipulation. It should, however, be noted that some other studies showed no difference in growth between enriched and barren housed pigs (20, 23, 54). Additionally, feed intake was also higher in the enriched pigs, which is in line with other studies (14, 17, 23). This could be related to the stimulation of exploration and/or easier access of pigs to the feeder in the enriched pens (87), which can facilitate feeder visits and, consequently, may increase feed intake (88), and therefore increase body weight gains.

## Conclusion

Enrichment with rooting substrates and extra space profoundly affected behavior and growth of pigs. The behavior of pigs that switched from barren to enriched pens or vice versa reflected, however, not only their actual environment, but was also influenced by their early life housing conditions. Generally, effects of enrichment, or a lack thereof, after the switch were more pronounced in pigs that had experienced a different early life condition, with pigs undergoing a downgrade displaying signs of frustration, whereas pigs exposed to an upgrade seemed to “catch up” by showing more exploration and play. Several effects of early life and current housing were stronger in pigs with a reactive personality. Thus, not only current housing conditions, but also a (mis)match with the early life environment affect behavior and growth of pigs. Results of this study imply that environmental enrichment throughout life is preferred, although application of enrichment at later life stages still improves pig welfare and performance. Moving pigs from enriched to barren environments, however, leads to frustration and should be avoided.

## Data Availability Statement

The datasets generated for this study are available on request to the corresponding author.

## Ethics Statement

The animal study was reviewed and approved by The Animal Care and Use Committee of Wageningen University & Research.

## Author Contributions

LL designed and did the animal experiment, did data analysis, and wrote the manuscript. IR participated in the animal experiment and helped with manuscript writing and revision. AM participated in the animal experiment and helped with data analysis and manuscript revision. BK helped with the data analysis and manuscript revision. JB designed the animal experiment, participated in data analysis, manuscript writing, and revision. All authors read and approved the final manuscript.

## Conflict of Interest

The authors declare that the research was conducted in the absence of any commercial or financial relationships that could be construed as a potential conflict of interest.
