# Supplementary material for: Effects of Early and Current Environmental Enrichment on Behavior and Growth in Pigs
Source: Front Vet Sci. 2020 Jun 4;7:268. doi: 10.3389/fvets.2020.00268 (PMC7287207; doi:10.3389/fvets.2020.00268)
Supplement: Supplementary file 1 [file Table_1.DOCX]

**Supplementary material**

Table 1: Means ± SEM of the percentages of time spent on behaviours for which a sex × day interaction was found in female and male pigs over observation days.

| Behaviour  (% of time) | Sex | Day 49 | Day 54 | Day 60 | Day 78 | Day 125 |
| --- | --- | --- | --- | --- | --- | --- |
| Inactivity | F | 52.8 ± 1.4^a^ | 56.4 ± 1.4^ab^ | 58.2 ± 1.2^b,x^ | 60.7 ± 1.2^b^ | 66.2 ± 1.3^c^ |
|  | M | 52.0 ± 1.4^a^ | 53.8 ± 1.4^a^ | 53.9 ± 1.3^a,y^ | 59.0 ± 1.6^b^ | 69.3 ± 1.2^c^ |
| Chewing | F | 11.0 ± 0.8^a^ | 10.9 ± 0.8^a^ | 8.2 ± 0.5^b^ | 8.2 ± 0.5^b^ | 7.6 ± 0.6^b^ |
|  | M | 10.4 ± 0.7^a^ | 10.3 ± 0.8^a^ | 8.1 ± 0.5^bc^ | 9.0 ± 0.7^ab^ | 6.1 ± 0.5^c^ |
| Chewing substrates | F | 8.1 ± 0.9^a^ | 8.0 ± 0.9^a^ | 5.4 ± 0.6^b^ | 5.2 ± 0.6^b^ | 5.1 ± 0.6^b^ |
|  | M | 7.4 ± 0.8^a^ | 7.5 ± 0.9^a^ | 5.4 ± 0.6^bc^ | 6.1 ± 0.7^ab^ | 4.1 ± 0.5^c^ |
| Manipulation | F | 2.4 ± 0.4^ab^ | 2.4 ± 0.4^a^ | 1.7 ± 0.3^ab^ | 2.0 ± 0.3^ab^ | 1.2 ± 0.2^b^ |
|  | M | 1.9 ± 0.3^a^ | 2.7 ± 0.3^b^ | 1.6 ± 0.2^a^ | 1.7 ± 0.2^a^ | 0.6 ± 0.1^c^ |
| Play | F | 1.5 ± 0.2^a^ | 1.2 ± 0.1^ab^ | 1.1 ± 0.1^a,x^ | 0.6 ± 0.1^bc^ | 0.5 ± 0.1^c^ |
|  | M | 1.6 ± 0.2^a^ | 1.4 ± 0.2^a^ | 1.9 ± 0.1^a,y^ | 0.7 ± 0.1^b^ | 0.2 ± 0.1^b^ |
| Aggression | F | 0.4 ± 0.1^x^ | 0.5 ± 0.1^x^ | 0.4 ± 0.1^x^ | 0.2 ± 0.1^x^ | 0.4 ± 0.1 |
|  | M | 1.4 ± 0.1^a,y^ | 1.3 ± 0.1^a,y^ | 1.2 ± 0.1^a,y^ | 0.9 ± 0.1^a,y^ | 0.4 ± 0.1^b^ |
| Mounting | F | 0.2 ± 0.1 | 0.1 ± 0.03 | 0.1 ± 0.04 | 0.05 ± 0.02 | 0.06 ± 0.02 |
|  | M | 0.8 ± 0.1 | 0.7 ± 0.1 | 0.5 ± 0.07 | 0.4 ± 0.08 | 0.4 ± 0.08 |

Only the behaviours affected by the sex × day interaction (p<0.05) are given, other behaviours are described in the text. ^abc^Indicate day effects within sex: Means lacking a common superscript letter a, b or c differ significantly (p<0.05) within the same sex. ^xy^Indicate sex effects within day: Means having a different superscript letter x, y differ significantly (p<0.05) within the same day.
